# Supplementary material for: Mouse pulmonary interstitial macrophages mediate the pro-tumorigenic effects of IL-9
Source: Nat Commun. 2022 Jul 1;13:3811. doi: 10.1038/s41467-022-31596-7 (PMC9249769; doi:10.1038/s41467-022-31596-7)
Supplement: Supplementary file 1 — Supplementary Information [file 41467_2022_31596_MOESM1_ESM.pdf]

Murine pulmonary interstitial macrophages mediate the pro-tumorigenic effects of IL-9

Yongyao Fu[1], Abigail Pajulas[1], Jocelyn Wang[1], Baohua Zhou[2], Anthony Cannon[1], Cherry Cheuk Lam Cheung[1], Jilu Zhang[1], Huaxin Zhou[3], Amanda Jo Fisher[3], David T Omstead[4], Sabrina Khan[4], Lei Han[2], Jean-Christophe Renauld[5], Sophie Paczesny[6], Hongyu Gao[7], Yunlong Liu[7], Lei Yang[2], Robert M Tighe[8], Paula Licona-Limón[9], Richard A. Flavell[10], Shogo Takatsuka[11], Daisuke Kitamura[11], Jie Sun[12], Basar Bilgicer[4], Catherine R Sears[3], Kai Yang[2], Mark H Kaplan[1,]*

Supplementary Figures and Tables

Supplementary Fig. 1

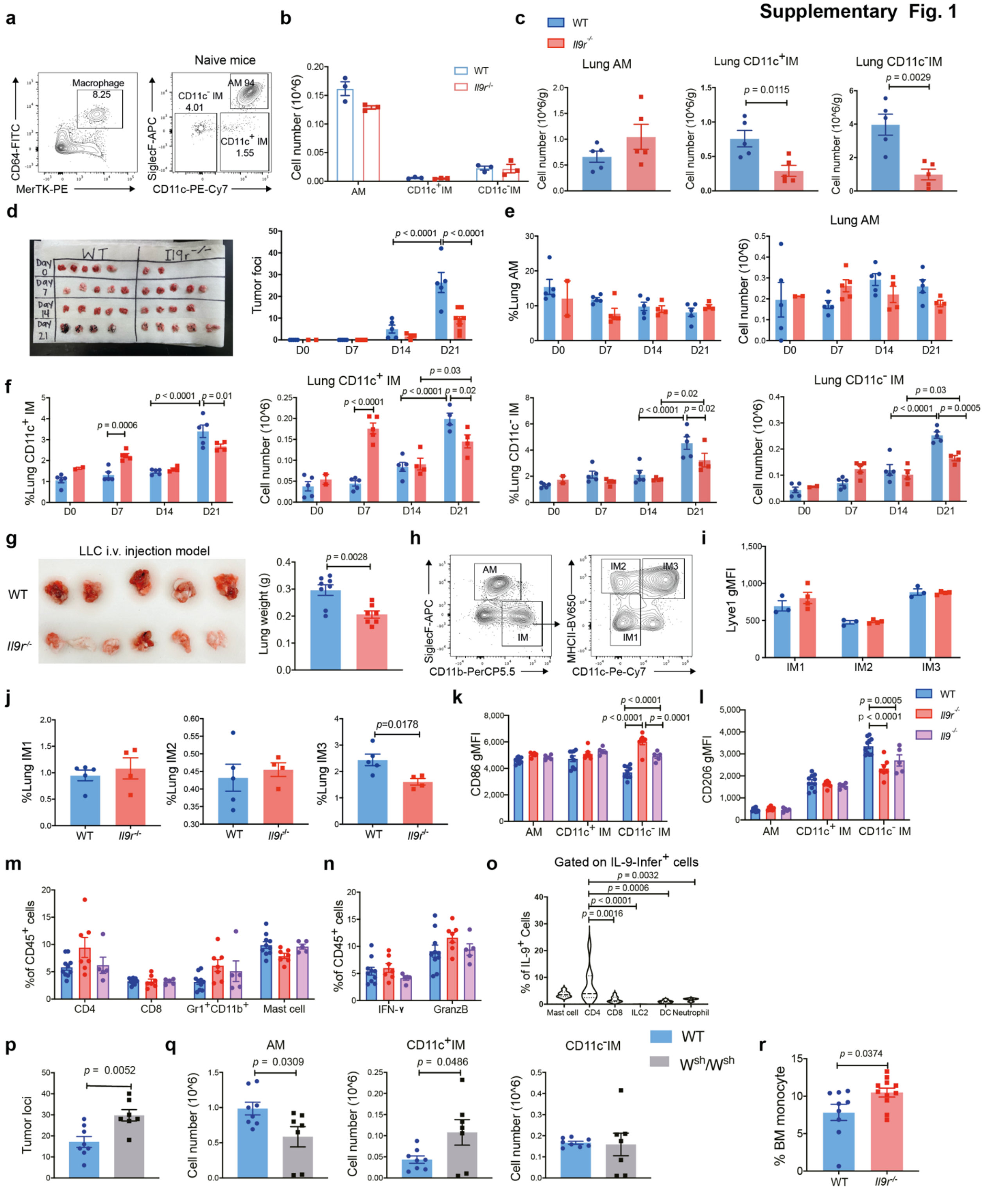

Supplementary Fig. 1. IL-9 promotes tumor growth by altering lung macrophage populations.
(a-b), Flow cytometric analysis of lung macrophage numbers from naïve mice (n = 3 mice).
(c), Flow cytometric analysis of lung macrophage numbers from tumor bearing mice (n =5 mice).
(d-f), Tumor bearing mice were analyzed after one, two or three weeks of tumor injection. Tumor growth (d) and lung macrophage numbers (e and f) were analyzed (n = 5 mice for WT group, n = 2 mice for d0 *Il9r*$^{-/-}$ group, n = 5 for d7 *Il9r*$^{-/-}$ group, n = 4 mice for d14 and d21 *Il9r*$^{-/-}$ group).
(g), Tumor growth and lung weight in LLC metastasis model (n = 8 mice for WT group, n = 7 mice *Il9r*$^{-/-}$ group).
(h), Dot plot analysis of IM subsets from B16 tumor bearing mice.
(i), Lyve1 expression in IM subsets from B16 tumor bearing mice (n = 3 mice for WT group, n = 4 mice *Il9r*$^{-/-}$ group).
(j), IM subset analysis from B16 tumor bearing mice (n = 5 mice for WT group, n = 4 mice *Il9r*$^{-/-}$ group).
(k-n), B16 melanoma cells were intravenously injected into the mice. CD86 expression (k) (n = 9 mice for WT group, n = 7 mice *Il9r*$^{-/-}$ group, n = 5 mice *Il9*$^{-/-}$ group) and CD206 expression (l) (n = 10 mice for WT group, n = 7 mice *Il9r*$^{-/-}$ group, n = 5 mice *Il9*$^{-/-}$ group), Immune cells and cytokine production from CD45^{+} cells (m-n) were analyzed by flow cytometry (n = 10 mice for WT group, n = 7 mice *Il9r*$^{-/-}$ group, n = 5 mice *Il9*$^{-/-}$ group).
(o), IL-9 reporter (Infer) mice were injected with B16 tumor, IL-9 producing cells were analyzed on day 21 (n = 8 mice for mast cell, DC and neutrophil, n = 9 mice for CD4 cell, n = 10 for CD8 and ILC2).
(p-q), Tumor growth (p) and lung macrophage numbers (q) were analyzed on day 21 (n = 8 mice for WT group, n = 7 mice for W^{sh}/W^{sh} group).
(r), Percentages of bone marrow monocytes from LLC intravenously injected tumor bearing mice were analyzed by flow cytometry (n = 9 mice for WT group, n = 11 mice *Il9r*$^{-/-}$ group).
Data are the mean ± SEM. Unpaired two-tailed Student t-test was used for comparison in c, j, p, q and r. Two-way ANOVA with Sidak's multiple comparisons was used for comparison in e, f, k and l. One-way ANOVA with a Dunnett's multiple comparison test was used for multiple comparisons in o.

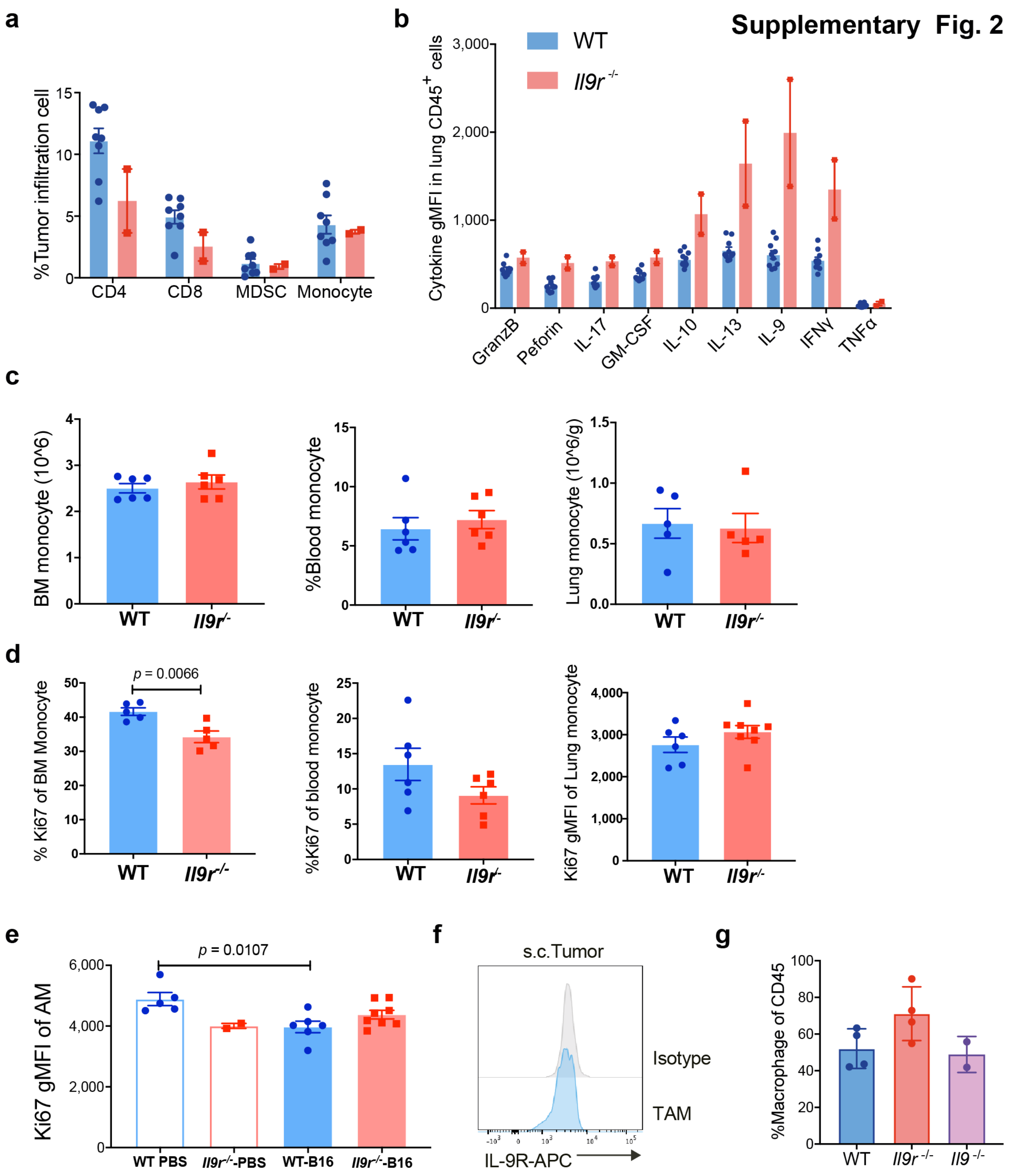
Supplementary Fig. 2
a
%Tumor infiltration cell
CD4
CD8
MDSC
Monocyte
b
WT
Il9r -/-
Cytokine gMFI in lung CD45+ cells
GranzB
Peforin
IL-17
GM-CSF
IL-10
IL-13
IL-9
IFNγ
TNFα
c
BM monocyte (10^6)
%Blood monocyte
Lung monocyte (10^6/g)
WT
Il9r-/-
d
% Ki67 of BM Monocyte
p = 0.0066
%Ki67 of blood monocyte
Ki67 gMFI of Lung monocyte
e
Ki67 gMFI of AM
p = 0.0107
WT PBS
Il9r-/--PBS
WT-B16
Il9r-/--B16
f
s.c.Tumor
Isotype
TAM
IL-9R-APC
g
%Macrophage of CD45
WT
Il9r -/-
Il9 -/-

Supplementary Fig. 2. IMs are the TAMs that respond to IL-9 and promote tumor growth
(a-b), LLC cells were injected to the lung, Tumor-infiltrating immune cells were analyzed by flow cytometry (a) (n = 9 mice for WT group, n = 2 mice for *Il9r*$^{-/-}$ group). (b), Cytokine production was analyzed (n = 9 mice for WT GranzB, Perforin, IL-17, and GM-CSF, n = 10 mice for WT IL-10, IL-13, IL-9, IFNγ and TNFα group, n = 2 mice *Il9r*$^{-/-}$ group).
(c), Monocytes from bone marrow, blood and lung were analyzed 21days after B16 tumor injection (n = 6 mice for groups in left and middle panel, n = 5 mice for groups in right panel).
(d), Ki67 expression in monocytes from bone marrow, blood and lung were analyzed 21 days after B16 tumor injection (n = 5 mice for groups in left panel, n = 6 mice for groups in middle panel, n = 6 for WT group and n = 8 mice for *Il9r*$^{-/-}$ group in right panel).
(e), Ki67 expression of lung AMs were analyzed 21 days after B16 tumor injection (n = 4 mice for WT PBS group, n = 2 mice for for *Il9r*$^{-/-}$ PBS group, n = 6 for WT-B-16 group and n = 8 mice for *Il9r*$^{-/-}$ - B16 group).
(f-g), IL-9R expression in TAMs (f) and macrophage percentage (g) from s.c. B16 tumor bearing mice (n = 4 mice for WT and *Il9r*$^{-/-}$ group, n = 2 mice for *Il9*$^{-/-}$ group).
Data are the mean ± SEM. Unpaired two-tailed Student t-test was used for comparison in d-e.

Supplementary Fig. 3

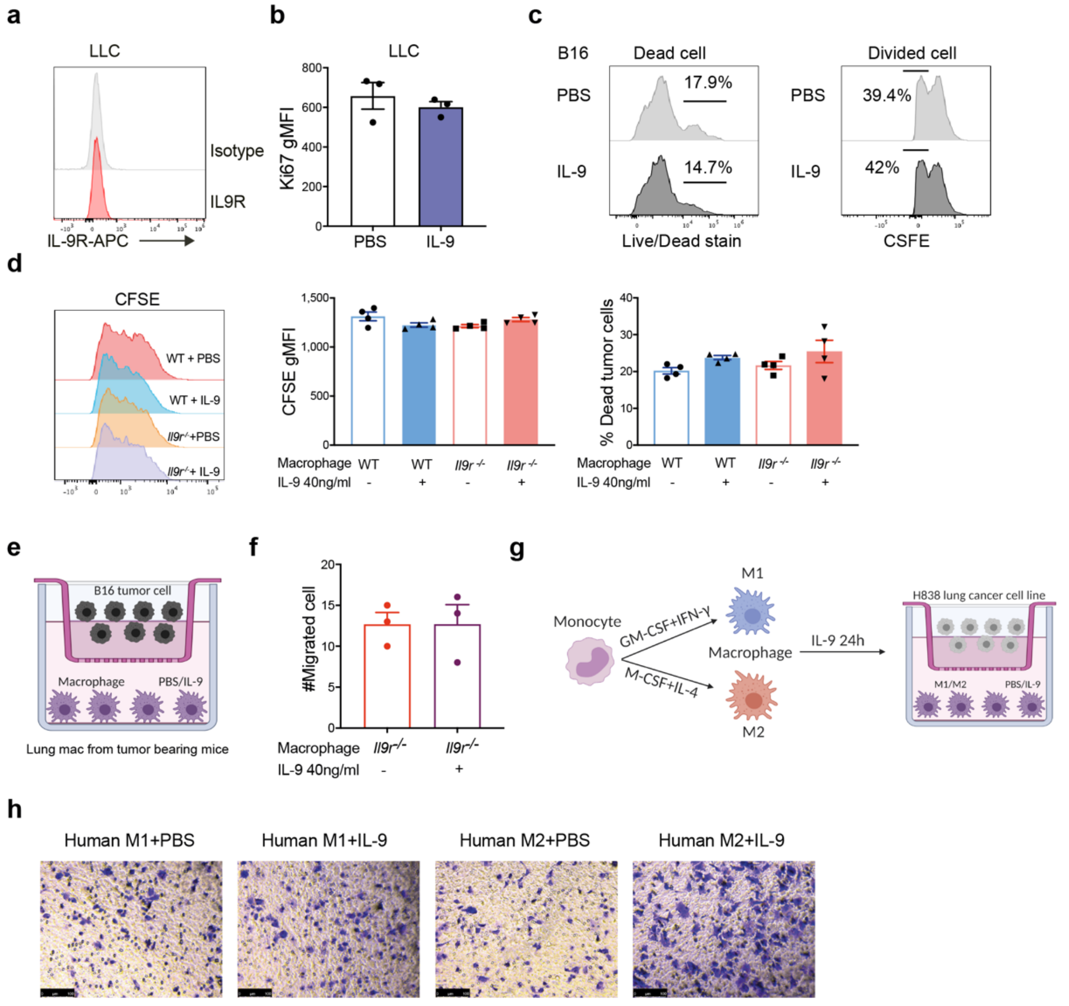

Supplementary Fig. 3. IL-9 promotes macrophage mediated tumor migration
(a), IL-9R expression on LLC cancer cell line was analyzed.
(b), LLC cancer cells were treated with IL-9 for 24hs. Ki67 expression was analyzed (n = 3 independent wells).
(c), B16 cells were treated with IL-9 for 48hs. Cell death and proliferation was analyzed.
(d), Total lung macrophages were isolated from LLC tumor bearing mice and cocultured with LLC cells for 72 hours. LLC cell proliferation and cell death were analyzed by flow cytometry (n = 4 mice).
(e-f), Total lung macrophages were isolated from tumor bearing mice and plated in the lower chamber of the transwell with or without IL-9, tumor cells were plated in the upper chamber (e).
(f), Migrated cells were visualized with crystal violet staining (n=3 mice).
(g-h), Human monocytes were cultured with GM-CSF or M-CSF for polarizing into M1 or M2 macrophage for 5 days before IFN-γ or IL-4 were respectively added to the cultures for 48hs. IL-9 was added for an additional 24 hours. The migration assay was performed (g) and human 838 cells were stained with crystal violet (h), Scale bar = 100 µm.

Data are the mean ± SEM. Unpaired two-tailed Student t-test was used for comparison in b and f. One-way ANOVA with a Dunnett's multiple comparison test was used for multiple comparisons in d.

Supplementary Fig. 4

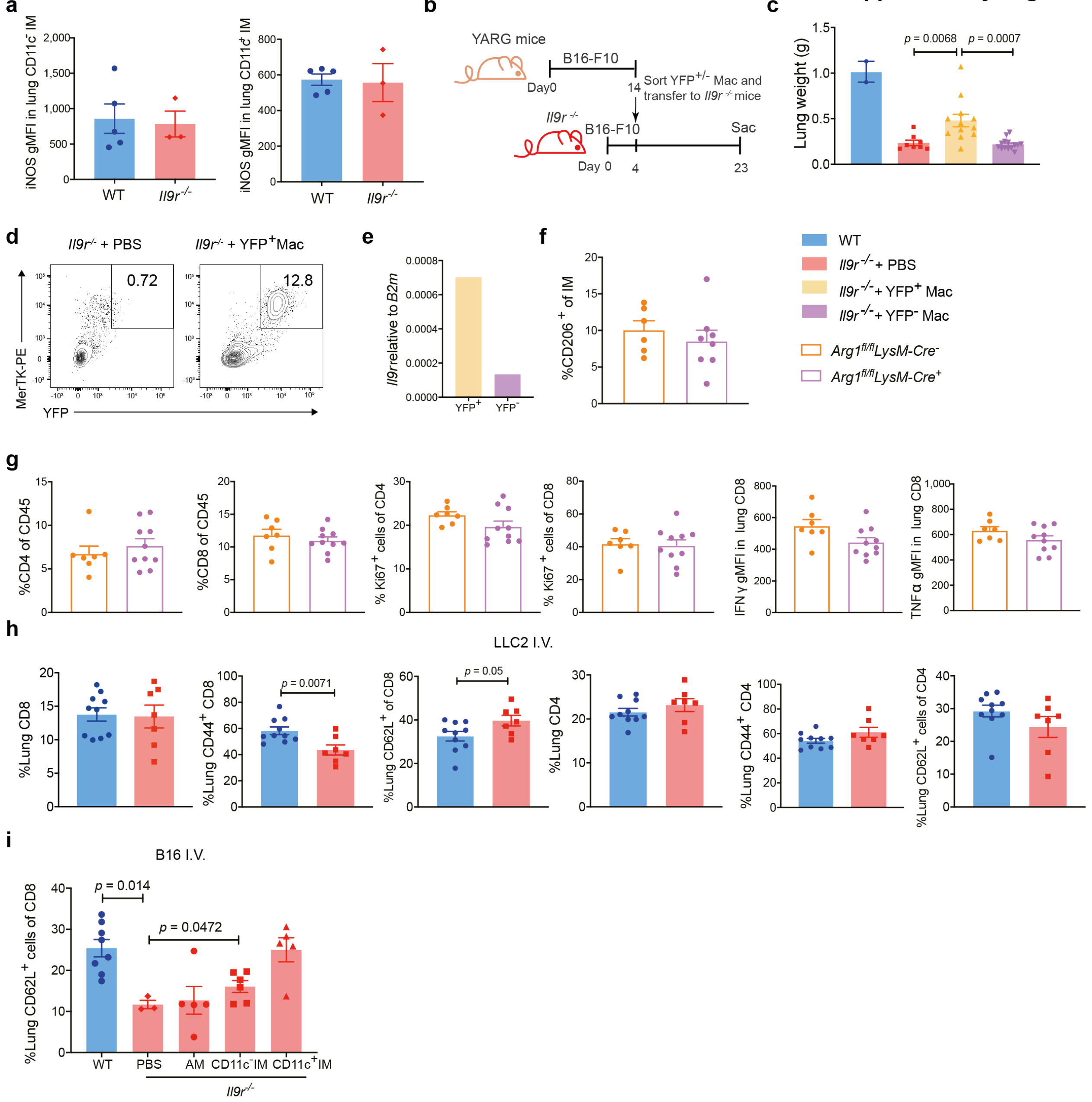

Supplementary Fig. 4. IL-9 impacts lung macrophage function by regulating Arg1 expression.

(a) iNOS expression from IMs were analyzed by flow (n= 5 mice for WT group, n = 3 for *Il9r*$^{-/-}$ group).

(b-e), YARG mice were injected with B16 cells, and lung Arg1$^{+/YFP}$ macrophages were sorted on day 14. Cells were intravenously injected into *Il9r*$^{-/-}$ mice 4 days after tumor injection (b). Lung weights were measured on day 23 (c) (n = 2 mice for WT group, n = 8 mice for *Il9r*$^{-/-}$ + PBS group,

n = 12 mice for *Il9r*$^{-/-}$ + YFP^{+} Mac group, n = 14 mice for *Il9r*$^{-/-}$ +YFP^{-} Mac group). Arg1 expression was analyzed in macrophages by flow cytometry (d). *Il9r* expression was analyzed from FACS sorted donor macrophages (e).

(f), CD206 expression in IMs from *Arg1*$^{fl/fl}$ *LysM-Cre*$^{+}$ mice or littermate control mice (n = 6 mice for *Arg1*$^{fl/fl}$ *LysM-Cre*$^{-}$ group, n = 8 mice for *Arg1*$^{fl/fl}$ *LysM-Cre*$^{+}$ group).

(g), Percentages of lung CD4 and CD8 T cells, Ki67 expression in T cells and cytokine expression from CD8 cells were analyzed by flow cytometry (n = 7 mice for *Arg1*$^{fl/fl}$ *LysM-Cre*$^{-}$ group and n = 10 mice for *Arg1*$^{fl/fl}$ *LysM-Cre*$^{+}$ group).

(h), Percentages of lung CD4 and CD8 T cells, CD44^{+} and CD62L^{+} T cells were analyzed by flow (n= 10 mice for WT group, n = 7 mice for *Il9r*$^{-/-}$ group).

(i), Macrophage adoptive transfer experiment was performed as shown in Fig. 5d. Lung CD62L+ CD8 T cells were analyzed (n =8 mice for WT group, n = 3 mice for PBS group, n = 5 mice for AM and CD11c^{+} IM groups, n = 6 mice for CD11c^{-} IM group).

Data are the mean ± SEM. One-way ANOVA with a Dunnett's multiple comparison test was used for multiple comparisons in c and i. Unpaired two-tailed Student t-test was used for comparison in h.

Supplementary Fig. 5

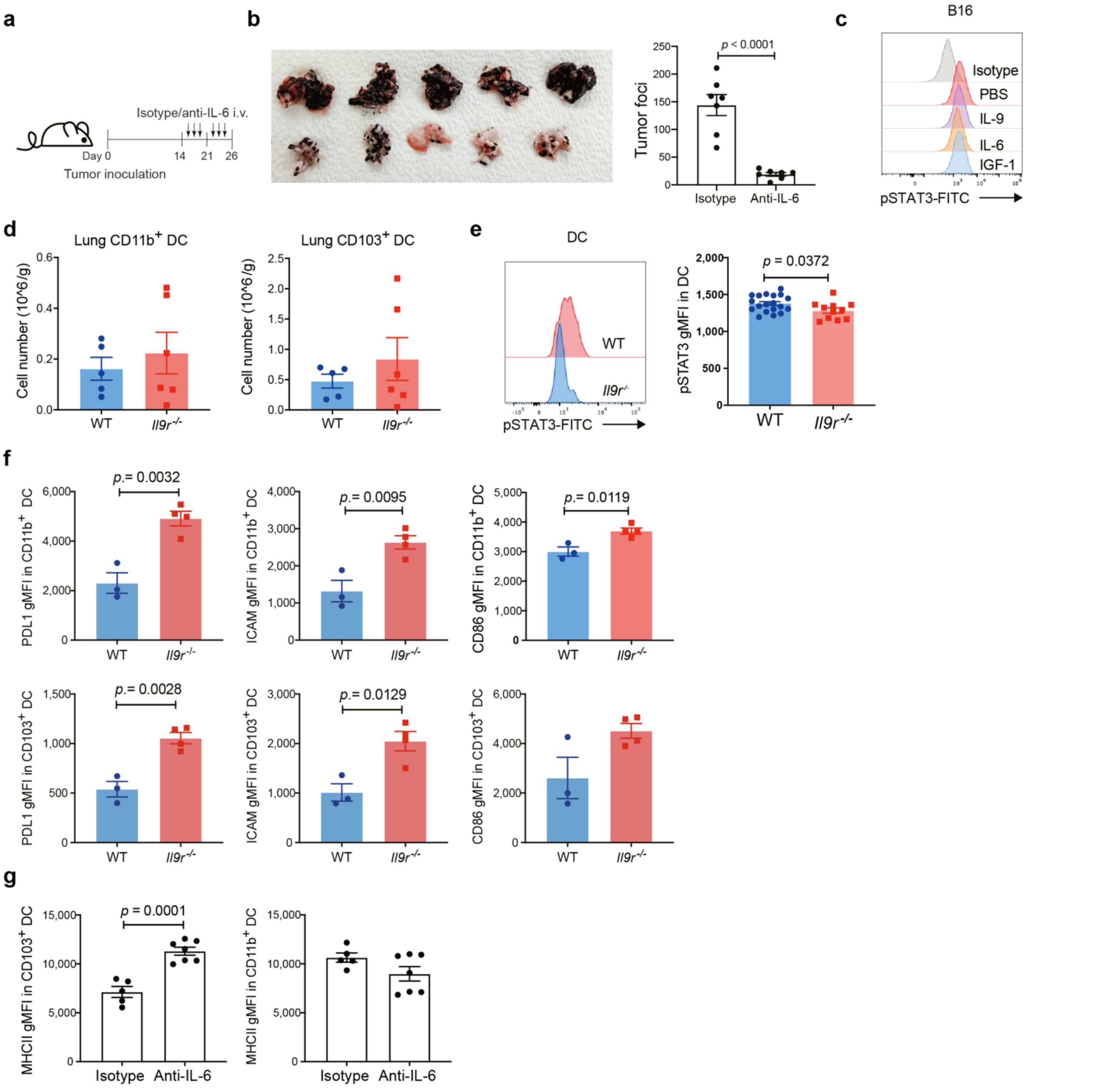

Supplementary Fig. 5. IL-9 induces IL-6 expression in Arg1 expressing IMs.
(a-b), WT tumor bearing mice were treated with isotype antibody or anti-IL-6 (a) and tumor growth was analyzed (b) (n = 7 mice).
(c), B16 cells were stimulated for 60 minutes and pSTAT3 expression was analyzed.
(d-f), DC numbers (d) (n = 5 mice for WT group, n = 6 for *Il9r*$^{-/-}$ group), pSTAT3 expression (e) (n = 19 mice for WT group, n = 11 mice for *Il9r*$^{-/-}$ group), and activation markers (f) (n = 3 mice for WT group, n = 4 mice for *Il9r*$^{-/-}$ group) were analyzed from B16 tumor bearing mice.
(g), Mice were treated as shown in b, MHCII expression from lung DCs was analyzed by flow cytometry (n = 5 mice for isotype group, n = 7 mice for anti-IL-6 group).
Data are the mean ± SEM. Unpaired two-tailed Student t-test was used for comparison.

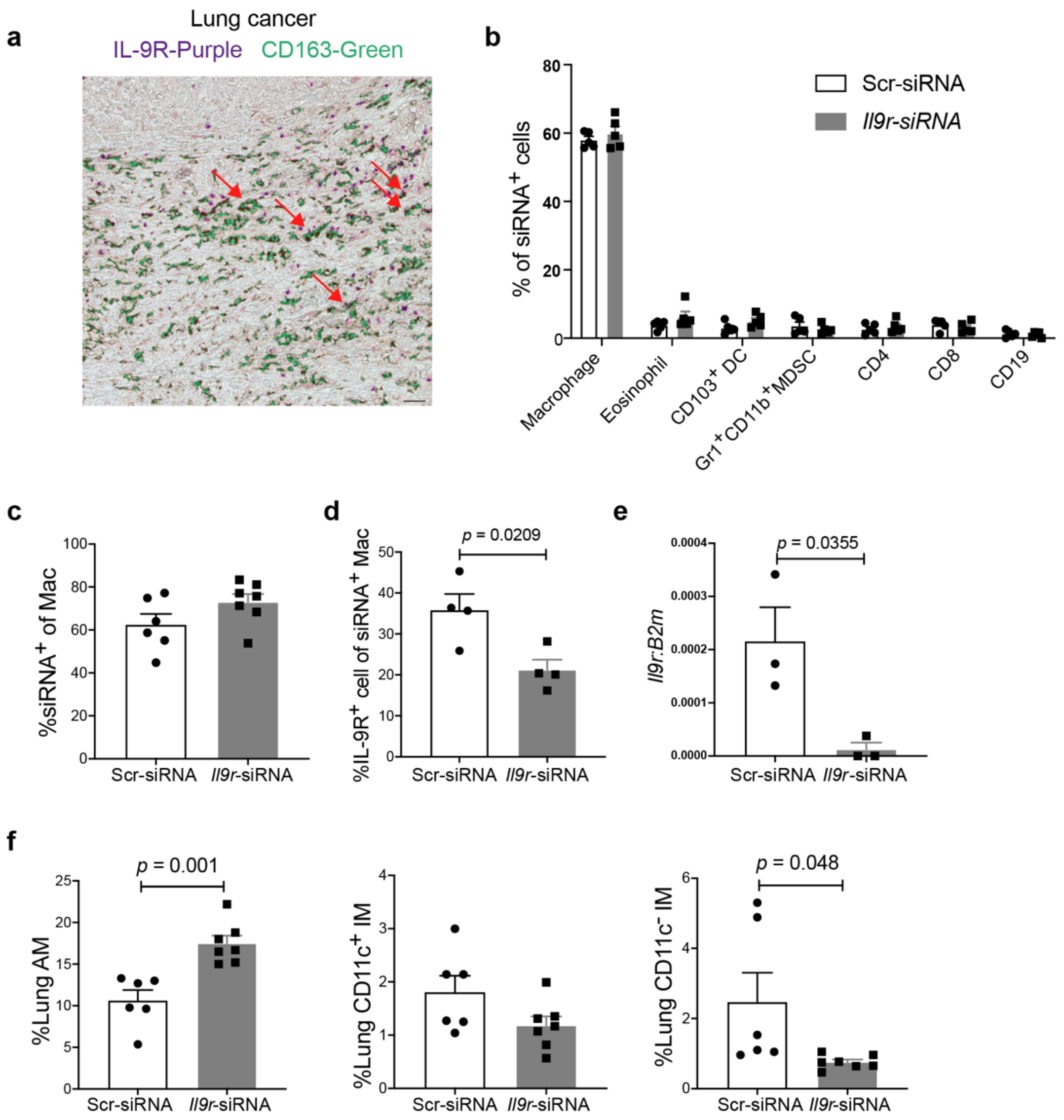

Supplementary Fig. 6. Therapeutic targeting of the IL-9-macrophage axis prevents lung cancer growth.

(a), Immunohistochemistry staining of CD163 and IL-9R in lung cancer patient tissue, Scale bar = 100 μm.

(b-f), WT mice were intravenously injected with B16 tumor cell line. Seven days after tumor inoculation, tumor-bearing mice were intravenously injected with nanoparticle-siRNA complexes every 72 hours. Scr/*Il9r*-siRNA was conjugated with Alexa Flour 555. Nanoparticles were tagged with SIRPα peptide. (b-c) Lung cells that had nanoparticle-siRNA complex uptake were analyzed by flow cytometry (n = 5 mice for groups in b, n = 6 mice for Scr-siRNA group and n = 7 for *Il9r*-siRNA group in c). (d), IL-9R expression in siRNA$^+$ (Alexa Flour 555) lung macrophages were analyzed by flow (n = 4 mice). (e), siRNA$^+$ (Alexa Flour 555) lung macrophages were sorted by gating on Alexa Flour 555$^+$ MerTK$^+$ CD64$^+$ live cells. Gene expression was analyzed (n = 3 mice). (f) Lung macrophages were analyzed by flow cytometry. (n = 6 mice for Scr-siRNA group and n

= 7 for *Il9r*-siRNA group in the middle and right panels). Data are the mean ± SEM. Unpaired two-tailed Student t-test was used for comparison.

Supplementary Fig. 7

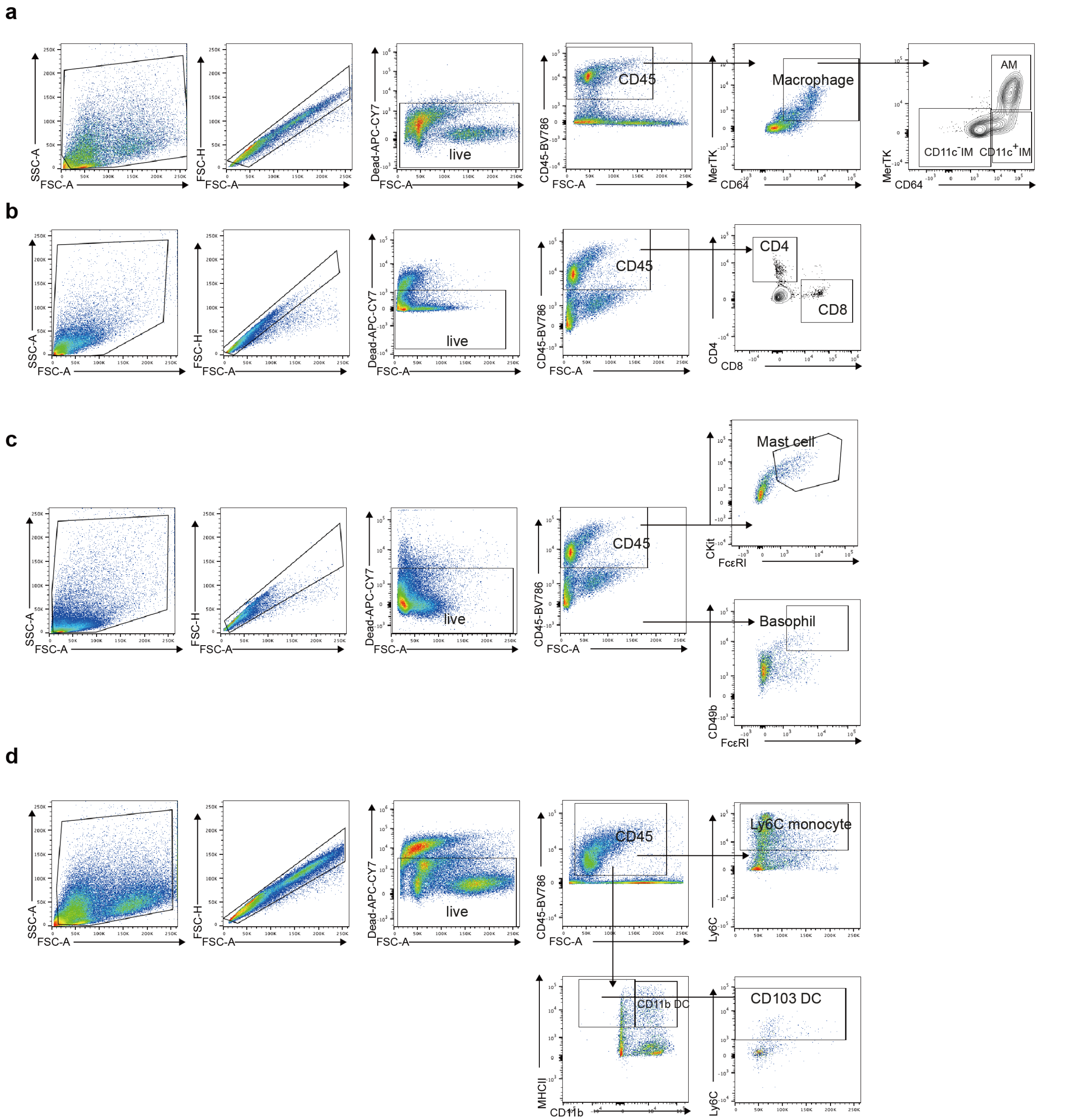

Supplementary Fig. 7. Gating strategies used for identifying different cell types

Gating strategies for identifying lung macrophages (a), T cells (b), mast cell and basophil (c), monocytes and DC (d). Strategy for macrophages was used in Figures 2c-f, h, i, k, Figure 4d, g, h, k, Figure 5b, c, Figure 7b-e, i, l, Figure 8e-j, Figure 9i, k, l and Supplementary Figures 1a-c, e-f, h, i-l, q-r, 2e-g, 4a, d, f, and 6c-f. T cell gating was used in Supplementary 4g-i. Monocyte gating was used in Supplementary 2c-d. DC gating was used in Supplementary 5c-g. All strategies were used for Figures 2c and f, 7l, and Supplementary figures 1m-o, 2a-b, and 6b.

Supplementary Tables

Supplementary Table 1. Lung cancer patient sample information related to Fig. 9c.

Description/diagnosis information	Gender	Age	Catalog Number
Lung, within normal limits	Female	74	CR561260
Lung, within normal limits	Female	76	CR563059
Lung, within normal limits	Male	72	CR559058
Lung, within normal limits	Female	76	CR562690
Lung, within normal limits	Male	46	CR562269
Lung, within normal limits	Female	55	CR560513
Lung, within normal limits	Male	45	CR562257
Lung, within normal limits	Male	72	CR559080
Lung, right upper lobe, adenocarcinoma of lung	Female	72	CR561124
Lung, carcinoma of lung, non-small cell	Male	65	CR561590
Lung, right upper lobe, adenocarcinoma of lung	Female	46	CR560308
Lung, carcinoma of lung, squamous cell, metastatic	Female	63	CR561521
Lung, carcinoma of lung, squamous cell	Male	57	CR562068
Lung, carcinoma of lung , squamous cell	Female	70	CR562209
Lung, right lower lobe, adenocarcinoma of lung	Male	72	CR562443
Lung, carcinoma of lung , squamous cell	Female	77	CR562025

Supplementary Table 2. Lung cancer patient sample information related to Fig. 9d.

Description/diagnosis information	Gender	Age
Healthy control	Female	53
Healthy control	Female	31
Healthy control	Female	24
Healthy control	Female	22
Healthy control	Female	47
Healthy control	Male	66
Healthy control	Female	65
Lung cancer	Male	70
Lung cancer	Female	64
Lung cancer	Female	31
Lung cancer	Female	47
Lung cancer	Female	53

Supplementary Table 3. Lung cancer patient sample information related to Fig. 9f-g.

Description/diagnosis information	Gender	Age	Catalog Number
Lung, within normal limits	Female	71	CS700238
Lung, within normal limits	Female	61	CS702878
Lung, within normal limits	Female	66	CS700243
Lung, within normal limits	Male	68	CS711408
Lung, within normal limits	Female	49	CS706500
Lung, within normal limits	Male	54	CS809755
Lung, within normal limits	Male	61	CS804028
Lung carcinoma of lung, squamous cell	Male	74	CS708748
Lung; Adenocarcinoma of lung	Male	52	CS707844
Lung; Carcinoma of lung, squamous cell	Male	81	CS704553
Lung; Carcinoma of lung, pleomorphic	Female	71	CS716372
Lung: right upper lobe; Carcinoma of lung, non-small cell, metastatic (EGFR+)	Male	51	CS708072
Lung: right middle lobe; Carcinoma of lung, small cell, metastatic	Female	70	CS813393
Lung; Carcinoma of lung, squamous cell, metastatic	Male	74	CS702498

Supplementary Table 4. Key resource table.

REAGENT or RESOURCE	SOURCE	IDENTIFIER	Dilution
Antibodies			
Anti-Mouse CD11b-PerCP-Cy5.5 (clone M1/70)	eBioscience	Cat#:45-0112-82	1:200
Anti-Mouse CD11c-PE-Cy7 (clone N418)	eBioscience	Cat#:25-0114-82	1:400
Anti-Mouse CD11c-BV650 (clone N418)	BioLegend	Cat#:117339	1:400
Anti-Mouse CD4-PE-Cy7 (clone GK1.5)	BioLegend	Cat#:100422	1:200
Anti-Mouse c-Kit-APC (clone 2B8)	BioLegend	Cat#:105811	1:200
Anti-Mouse CD49b-PE (clone HMα2)	BioLegend	Cat#:103506	1:200
Anti-Mouse Ki67- PE-eFluor 610 (clone SolA15)	Invitrogen	Cat#:61-5698-82	1:400
Anti-Mouse CCR7-Alexa Fluor 700 (clone 4B12)	eBioscience	Cat#:56-1971-82	1:200
Anti-Mouse Arg1-APC (clone A1exF5)	Invitrogen	Cat#:17-3697-82	1:200
Anti-Mouse IL-6-PE (clone MP5-20F3)	BD Biosciences	Cat#:554401	1:200
Anti-Mouse CD206-APC (clone C068C2)	BioLegend	Cat#:141708	1:200
Anti-Mouse CD45.1-BV786 (clone A20)	BD Biosciences	Cat#:740889	1:200
Anti-Mouse CD45.1-PerCP5.5 (clone A20)	BioLegend	Cat#:110728	1:200
Anti-Mouse CD45.2-PE-Cy7 (clone 104)	Invitrogen	Cat#:25-0454-82	1:200
Anti-Mouse CD115-APC (clone AFS98)	BioLegend	Cat#:135510	1:200
Anti-Mouse CCR2-BV786 (clone 475301)	BD Biosciences	Cat#:747966	1:200
Anti-Mouse I-A/I-E-BV421 (clone M5/114.15.2)	BioLegend	Cat#:107631	1:400
Anti-Mouse CD103-BV421 (clone M290)	BD Biosciences	Cat#:557495	1:200
Anti-Mouse Ly-6C-BV510 (clone HK1.4)	BioLegend	Cat#:128033	1:400
Anti-Mouse FcεR1-PE-Cy7 (clone MAR-1)	BioLegend	Cat#:134317	1:200
Anti-Mouse Foxp3-FITC (clone MF23)	BD Biosciences	Cat#:560403	1:200
Anti-Mouse IFN-γ-PerCP-Cy5.5 (clone XMG 1.2)	eBioscience	Cat#:45-7311-82	1:200
Anti-Mouse IL-17A-PE-Cy7 (clone eBio17B72)	eBioscience	Cat#:25-7177-82	1:200
Anti-Mouse Ly6G-FITC (clone RB6-8C5)	BD Biosciences	Cat#:553126	1:200
Anti-Mouse SiglecF-BV605 (clone E50-2440)	BD Biosciences	Cat#: 740388	1:200
Anti-Mouse SiglecF-AF647 (clone E50-2440)	BD Biosciences	Cat#: 562680	1:200
Anti-Mouse CD64-FITC (clone X54-5/7.1)	BioLegend	Cat#: 139316	1:200
Anti-Mouse CD64-BV421 (clone X54-5/7.1)	BioLegend	Cat#: 139309	1:200
Anti-Mouse CD64-BV711 (clone X54-5/7.1)	BioLegend	Cat#: 139311	1:200
Anti-Mouse MerTK-PE (clone 2B10C42)	BioLegend	Cat#: 151506	1:200
Anti-Mouse MerTK-APC (clone 2B10C42)	BioLegend	Cat#: 151508	1:200
Anti-Mouse Gr1-APC (clone RB6-8C5)	BioLegend	Cat#: 108412	1:800
Anti-Human CD14-AF-700 (clone HCD14)	BioLegend	Cat#: 325605	1:200
Anti-Human IL-9R-PE (clone AH9R7)	BioLegend	Cat#: 310403	1:100
Biotin anti-mouse CD170 (SiglecF) Antibody (S17007L)	BioLegend	Cat#: 155512	1:200
Anti-Mouse Lyve1-APC	Thermo Fisher	Cat#: PA5-22782	1:200
Anti-Mouse Ly6G-PE-TexRed	Thermo Fisher	Cat#: 61-9668-82	1:200
Anti-Mouse MHCII-BV650	BC Bioscience	Cat#: 563415	1:400
Anti-Mouse IL-9R-APC	BioLegend	Cat#: 158806	1:100

Anti-Mouse PD-L1/CD274-BV650 (clone 10F.9G2)	BioLegend	Cat# 124336	1:200
Anti-Mouse CD86-FITC (clone GL-1)	BioLegend	Cat# 105006	1:200
Anti-Mouse ICAM-1/CD102-BV605 (clone 3C4(mIC2/4))	BD Bioscience	Cat# 740346	1:200
APC Rat IgG1, κ Isotype Ctrl	BioLegend	Cat#: 400412	1:200
FITC Rat Anti-Mouse IgG1	BD Biosciences	Cat#: 553443	1:200
FITC anti-STAT3 Phospho (Tyr705)	BioLegend	Cat#: 651020	1:200
Anti-Human IL-9R Polyclonal Antibody	Thermo Fisher	Cat#: PA5-84652	1:50
Anti-Human CD68 antibody [PG-M1]	Abcam	Cat#: Ab783	1:50
Anti-Mouse CD31-AF594	BioLegend	Cat#: 102520	1:50
Chemicals, Peptides, and Recombinant Proteins			
Collagenase D	Sigma-Aldrich	Cat#:11088882001	
Monocyte Isolation Kit (BM), mouse	Miltenyi Biotec	Cat#:130-100-629	
CD14 MicroBeads UltraPure, human	Miltenyi Biotec	Cat#:130-118-906	
Anti-Mer antibodies, mouse	Miltenyi Biotec	Cat#:130-107-477	
Recombinant mouse IL-9 (carrier free) - 25ug	BioLegend	Cat#:556004	
Recombinant Murine IL-4 20ug	Peprotech	Cat#:214-14	
Recombinant Human IL-9 Protein	R&D systems	Cat#:209-ILB-010	
Recombinant Human IL-4	Peprotech	Cat#:200-04	
Recombinant Human M-CSF	Peprotech	Cat#:300-25	
Recombinant Human GM-CSF	Peprotech	Cat#:300-03	
Recombinant Mouse IL-6	Peprotech	Cat#:216-16	
Human IGF-I Recombinant Protein	Thermo Fisher	Cat#: PHG0071	
InVivoMAb anti-mouse IL-6 (clone MP5-20F3)	BioXCell	Cat#:BE0046	
InVivoMAb rat IgG1 isotype control	BioXCell	Cat#:BE0088	
qScript™ cDNA Synthesis Kits	Quantabio	Cat#:101414-100	
TRIzol	Invitrogen	Cat#:15596018	
eBioscience™ Permeabilization Buffer (10X)	Thermo Fisher	Cat#:00-8333-56	
Hematoxylin	Dako	Cat#:S3301	
Citrate buffer	Sigma	Cat#:C9999-100ml	
CAS Block	ThermoFisher	Cat#: 008120	
DAPI – Fluoromount-G	SouthernBiotech	Cat#: 0100-20	
10% normal goat blocking serum	Thermo Fisher	Cat#:50197z	
AEC subtract	Vector laboratories	Cat#:SK-4200	
Critical Commercial Assays			
RNeasy Mini Kit	QIAGEN	Cat#: 74104	
Arginase Activity Assay Kit	Millipore Sigma	Cat#: MAK112-1KT	
Foxp3 / Transcription Factor Staining Buffer Set	eBioscience	Cat#: 00-5523-00	
eBioscience™ Permeabilization Buffer (10X)	eBioscience	Cat#: 00-8333-56	
ELISA MAX™ Deluxe Set Human IL-9 Kit	Biolgend	Cat#: 434704	
ELISA MAX™ Deluxe Set Human IL-6 Kit	Biolgend	Cat#: 430504	
ELISA MAX™ Deluxe Set Mouse IL-6 Kit	Biolgend	Cat#: 431304	
Experimental Models: Cell Lines			

B16-F10	Laboratory of Dr. Dario Vignali and Dr. Kai Yang	
LLC (LL/2-Luc2)	ATCC	CRL-1642-LUC2
H838	ATCC	CRL-5844
Oligonucleotides		
Primers for mRNA expression, see Supplementary Table 6		
Il9r-siRNA for Figure 8	ThermoFisher	Cat#S201546
Scr-siRNA for Figure 8	ThermoFisher	Cat#4457289
Software and Algorithms		
GraphPad Prism V_8.0 and V_7.0	GraphPad Software	http://www.graphpad.com
FlowJo (version 10.7.1)	LLC	http://www.flowjo.com
ImageJ V1.53a	NIH software	https://imagej.nih.gov/ij/
R language	The R Foundation	https://www.r-project.org/

Supplementary Table 5. Probes for mRNA expression related to Key resource table.

DESCRIPTION	IDENTIFIER
Mouse *β2m*	Cat#:Mm00437762_m1
Mouse *Arg1*	Cat#:Mm00475988_m1
Mouse *Il9r*	Cat#:Mm00434313_m1
Human *B2M*	Cat#:Hs06637353_s1
Human *IL6*	Cat#:Hs00174131_m1
Human *IL9R*	Cat#:Hs01108522_m1
